# Generation and Characterization of α9 and α10 Nicotinic Acetylcholine Receptor Subunit Knockout Mice on a C57BL/6J Background

**DOI:** 10.3389/fnins.2017.00516

**Published:** 2017-09-21

**Authors:** Barbara J. Morley, David F. Dolan, Kevin K. Ohlemiller, Dwayne D. Simmons

**Affiliations:** ^1^Center for Sensory Neuroscience, Boys Town National Research Hospital Omaha, NE, United States; ^2^Kresge Hearing Research Institute, University of Michigan Ann Arbor, MI, United States; ^3^Department of Otolaryngology, Washington University St. Louis, MO, United States; ^4^Biology, Baylor University Waco, TX, United States

**Keywords:** nicotinic acetylcholine receptor, auditory brainstem response, distortion product otoacoustic emissions, efferent strength

## Abstract

We generated constitutive knockout mouse models for the α9 and α10 nicotinic acetylcholine receptor (nAChR) subunits by derivation from conditional knockouts by breeding with CRE deleter mice. We then backcrossed them onto a C57BL/6J genetic background. In this manuscript, we report the generation of the strains and an auditory phenotypic characterization of the constitutive α9 and α10 knockouts and a double α9α10 constitutive knockout. Although the α9 and α10 nAChR subunits are relevant to a number of physiological measures, we chose to characterize the mouse with auditory studies to compare them to existing but different α9 and α10 nAChR knockouts (KOs). Auditory brainstem response (ABR) measurements and distortion product otoacoustic emissions (DPOAEs) showed that all constitutive mouse strains had normal hearing. DPOAEs with contralateral noise (efferent adaptation measurements), however, showed that efferent strength was significantly reduced after deletion of both the α9 and α10 subunits, in comparison to wildtype controls. Animals tested were 3–8 weeks of age and efferent strength was not correlated with age. Confocal studies of single and double constitutive KOs showed that all KOs had abnormal efferent innervation of cochlear hair cells. The morphological results are similar to those obtained in other strains using constitutive deletion of exon 4 of α9 or α10 nAChR. The results of our physiological studies, however, differ from previous auditory studies using a α9 KO generated by deletion of the exon 4 region and backcrossed onto a mixed CBA/CaJ X 129Sv background.

## Introduction

The α9 and α10 nicotinic acetylcholine receptor (nAChR) subunits are members of the subfamily I, epithelial nicotinic receptor gene family. The α9 and α10 subunits assemble as a heteromeric pentamer when heterologously expressed in *Xenopus* oocytes and *in vivo* in cochlear hair cells (reviewed in Elgoyhen and Katz, 2012; Goutman et al., 2015). The α9α10 nAChR is atypical because both subunits are α subunits. It was proposed that the α10 subunit might act as a structural subunit because α10 is necessary but not sufficient to maintain wildtype electrophysiological activity in heterologous systems and cochlear hair cells (Plazas et al., 2005). There is no evidence that either the α9 or the α10 subunit is assembled with another member of the nAChR gene family in hair cells. Although other nAChR subunits were localized in the cochlea, there was no evidence of expression of other neuronal subunits in hair cells using *in situ* hybridization (Morley et al., 1998).

The apparent reason why the avian but not mammalian α10 can form a functional homomeric receptor when heterologously expressed is that the mammalian α10 subunit underwent positive selection pressure and synonymous substitutions occurred in the complementary face of the α10 subunit (Lipovsek et al., 2014; Boffi et al., 2017). The contribution of mammalian α9 and α10 to complementary binding sites is nonequivalent (Boffi et al., 2017), thus rendering the mammalian α10 incapable of forming a homomer that conducts current (Franchini and Elgoyhen, 2006; Lipovsek et al., 2012). In a recent study, it was demonstrated, however, that both α9 and α10 subunits contribute to the principal component of the agonist binding site, contradicting the hypothesis that the α10 is a structural subunit, but confirming that the integrity of both subunits is necessary for the wildtype receptor function in mammals (Sgard et al., 2002; Vetter et al., 2007; Taranda et al., 2009; Boffi et al., 2017).

The α9 and α10 subunits were first identified in in the olfactory epithelium and embryonic and adult hair cells of the vertebrate inner ear (Elgoyhen et al., 1994, 2001; Hiel et al., 1996; Simmons and Morley, 1998, 2011; Morley and Simmons, 2002). Studies of cochlear hair cells in α9 or α10 knockouts have largely dominated experimentation of α9α10 *in vivo* pharmacology and physiology (Katz et al., 2000; Verbitsky et al., 2000; reviewed in Elgoyhen and Katz, 2012), in part because the cholinergic innervation of cochlear outer hair cells (OHC) was already well-known (e.g., Erostegui et al., 1994; Nenov et al., 1996; reviewed in Elgoyhen and Katz, 2012) and in part because their localization was generally thought to be limited to sensory hair cells. Although not reported to exist in brain, α9 and α10 subunits are now known to be widely distributed. For example, one or both subunits have been localized in the pituitary (Elgoyhen et al., 1994, 2001; Luebke et al., 2005), retina (Smith et al., 2014), Scarpa's ganglion (Luebke et al., 2005), immune cells (Peng et al., 2004; Hao et al., 2011; Koval et al., 2011; St-Pierre et al., 2016), brain and breast tumors (Russo et al., 2014; Spina et al., 2016), skin keratinocytes (Chernyavsky et al., 2007; Grau et al., 2007), colon (Bader and Diener, 2015), renal allografts (Meixner et al., 2014), and osteoblasts (Zablotni et al., 2015).

It is typically inferred that α10 is present whenever α9 is detected and that they always assemble, but there are data that suggest that α7 and α10 could form a receptor (Lips et al., 2006; Morley et al., 2017). Although α9-containing nAChRs are ionotropic, they may also mediate metabolic signaling (Richter et al., 2016; Backhaus et al., 2017; Zakrzewicz et al., 2017). There is *in vivo* evidence that α9α10 can also exist in at least two stoichiometries, (α9)_3_(α10)_2_ and (α9)_2_(α10)_3_, and that ACh has an additional low sensitive binding site located at the α9-α9 interface (Indurthi et al., 2014). It is possible that different stoichiometries exist in different tissues *in vivo* (Indurthi et al., 2014). Thus, there are unanswered questions and there is a need for further study of the α9 and α10 subunits in diverse tissues.

In the cochlea, the α9α10 nAChR mediates the effects of the medial olivocochlear (MOC) efferent pathway. This pathway regulates the auditory afferent input to the brain by reducing the gain produced by OHC via release of acetylcholine (Fuchs, 2014; Guinan, 2014). Earlier studies of mice lacking either a functional α9 or α10 subunit showed that the MOC terminals were larger than normal and that there was a reduction in the MOC suppression of cochlear responses (Vetter et al., 1999, 2007). Although significant, the phenotype produced in these knockouts was much less severe than previous studies where MOC fibers were ablated. Neither the α9 or α10 deletion has any effect on cochlear base line sensitivity, or demonstrate a decrease in tone detection and intensity discrimination in quiet and continuous background noise, as has been shown for mice with MOC pathway lesions (May et al., 2002; Vetter et al., 2007). Deletion of both α9 and α10 subunits may be necessary to observe a more severe phenotype.

In this manuscript we report the generation of conditional and constitutive KOs for the α9 and α10 subunits of the nAChR gene family on a C57BL/6J background. In the α9 KO, exons 1-2 were deleted and in the α10 KO, exons 1–3 were deleted. ABRs and DPOAEs in the α9 and α10 subunit constitutive KOs were normal. Although there were no significant differences in the ABR responses to noise, deletion of both genes resulted in less suppression of DPOAEs with contralateral noise. Confocal analysis of the cochlear innervation of hair cells showed aberrant innervation patterns.

## Materials and methods

### Generation of the α9 mutants (knockouts)

The cloning and the initial generation of the mice were conducted by Genoway, Inc. (Lyon, France). 10.5 kb of mouse genomic DNA encompassing the α9 subunit gene region surrounding exons 1 and 2 on chromosome 5 were isolated from the 129Sv/Pas genetic background and fully sequenced.

**Figure 1:**
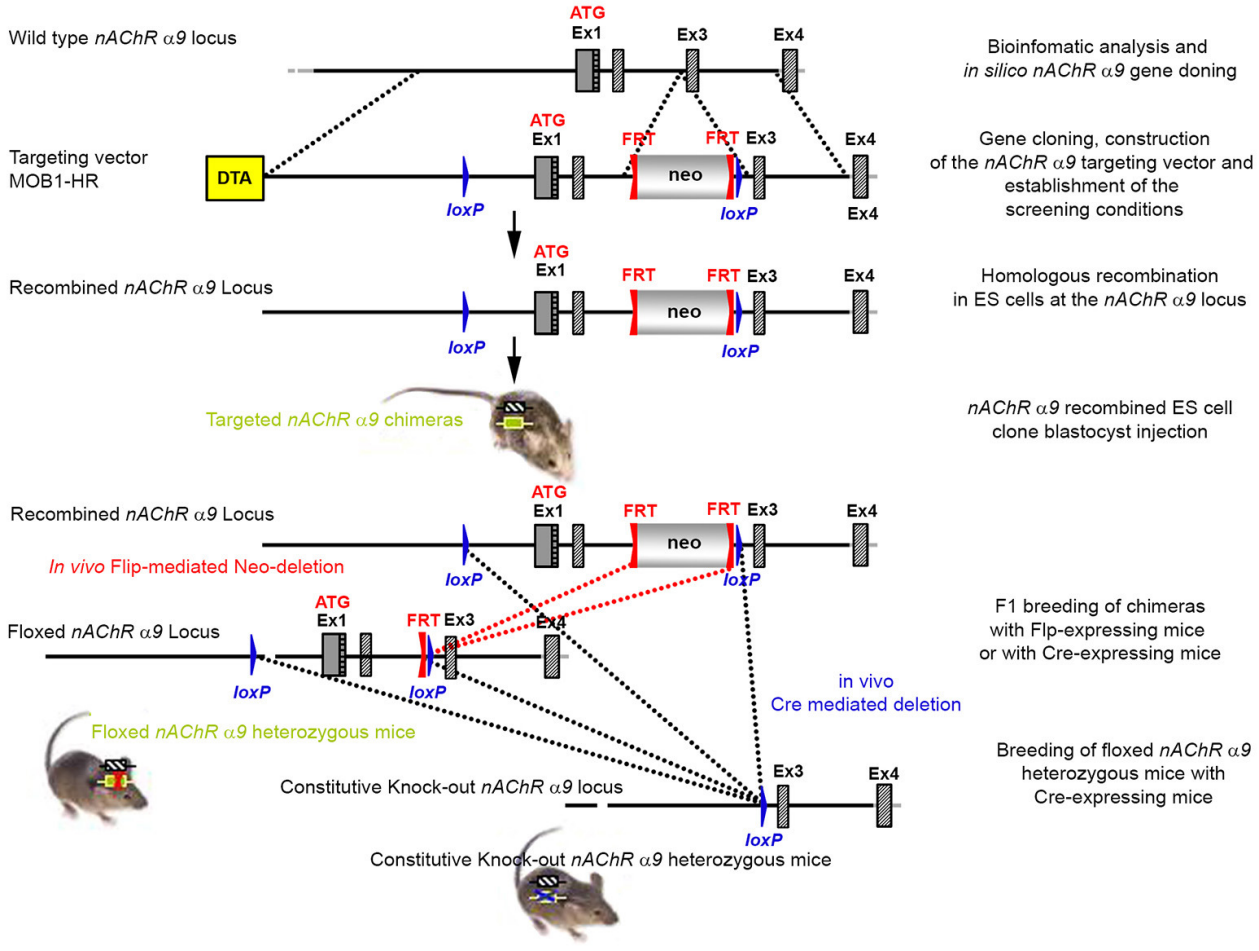
The targeting strategy for the generation of the *CHRNA9* knockout. Boxes represent exons. Solid line represents intronic sequences. LoxP and FRT elements are shown as blue and red triangles, respectively. Neo and DTA boxes represent the neomycin positive selection cassette and the Diphtheria Toxin A negative selection cassette. The constitutive KO was generated by breeding the floxed nAChR α9 heterozygous mice with Cre-expressing mice.

The targeting strategy for the generation of the *CHRNA9* knockout is shown in Figure 1. Exons 1 and 2 of *CHRNA9* were deleted and their flanking intronic sequences were replaced by a validated FRT-neomycin-FRT-*loxP* cassette at the 3′ end and a single *loxP* site in the 5′ direction, which effectively eliminated the translation of any truncated form of the gene. The distal l*oxP* site was positioned upstream of exon 1 within the promoter sequence. A 7,315 bp positive control (MPB1-C+) and 16,500 bp targeting vector (MOB1-HR) were constructed and validated. Neomycin (neo) was used as the positive selection marker. The targeting vector used the Diphtheria Toxin A (DTA) as a negative selection marker. Functionality of *the loxP* and FRT sites was confirmed by transforming *E. coli* with the targeting vector and PCR using the reverse primer GGACCCACAGAATGAACTGAGTTGACC and forward primer GGTACGCATCGTGCCAAGTTTGG, which were located downstream of the neomycin cassette and upstream of the *loxP* site, respectively.

**Figure 2:**
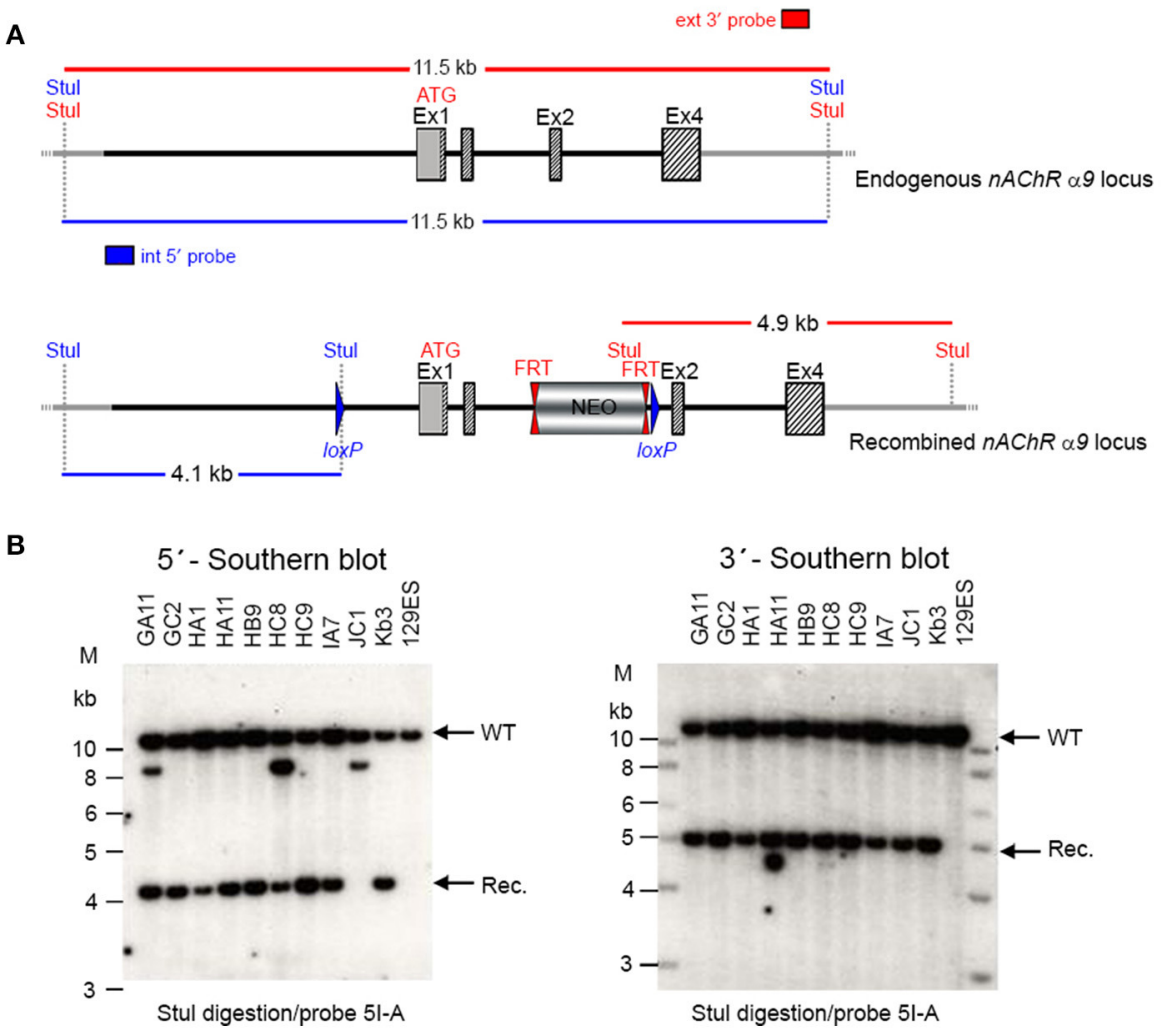
**(A)** The schemata for the endogenous and recombined a9 nAChR loci. **(B)** The Southern blot analysis for 5′ and 3′ homologous recombination in ES cells (α9 nAChR). The genomic DNA of the 10 tested ES cell clones was compared with wild type DNA from 129ES. Genomic DNA was digested with StuI. The digested DNA was blotted on nylon membrane and hybridized with either an internal 593 bp 5′ probe (5I-A) or the external 378 bp 3′ probe (3E-J) to screen for 5′ and 3′ homologous recombination. The internal 593 bp 5′ probe was generated by PCR on genomic DNA using the primer pair CTAAGGTGGCAGCAGAATGGAGTTCC/GCACGGGTACAAGGGTGAAGATGC. The 5′ probe hybridized downstream of the 5′ homology sequence of the targeting vector, which also allowed detection of the ES cell clones in which the targeting vector was integrated in a non-homologous manner. The 378 external bp 3′ probe was generated by PCR on genomic DNA using the primer pair ACAGAATGCACTTTGAAAAGTC/CTCAAGAAGCAAAAAACAGC. The expected size of the WT allele was 11,458 bp and the recombined allele 4,138 bp with the 5′ probe and 11,458 and 4,911 bp, respectively, with the 3′ probe. Cell clones GC2, HA11, HB9, HC9, IA7, and KB3 all showed WT and recombined signals of similar intensity and were chosen for blastocyst injection.

The targeting vector was linearized with Nrul and the construct was transfected into ES cells according to standard electroporation procedures. Two hundred and nine isolated clones were initially screened by PCR to test for homologous recombination at the 3′ end with the primer sequences CCAGTCATAGCCGAATAGCCTCTCCAC/TGTCGAAGGGGAAATAGGTGACATCC. Ten positive clones were expanded and the 3′ homologous recombination confirmed for the 10 clones with PCR. Six correctly targeted ES clones were verified by Southern blot at the 5′ and 3′ sides (Figure 2) and selected for blastocyst injection.

**Figure 3:**
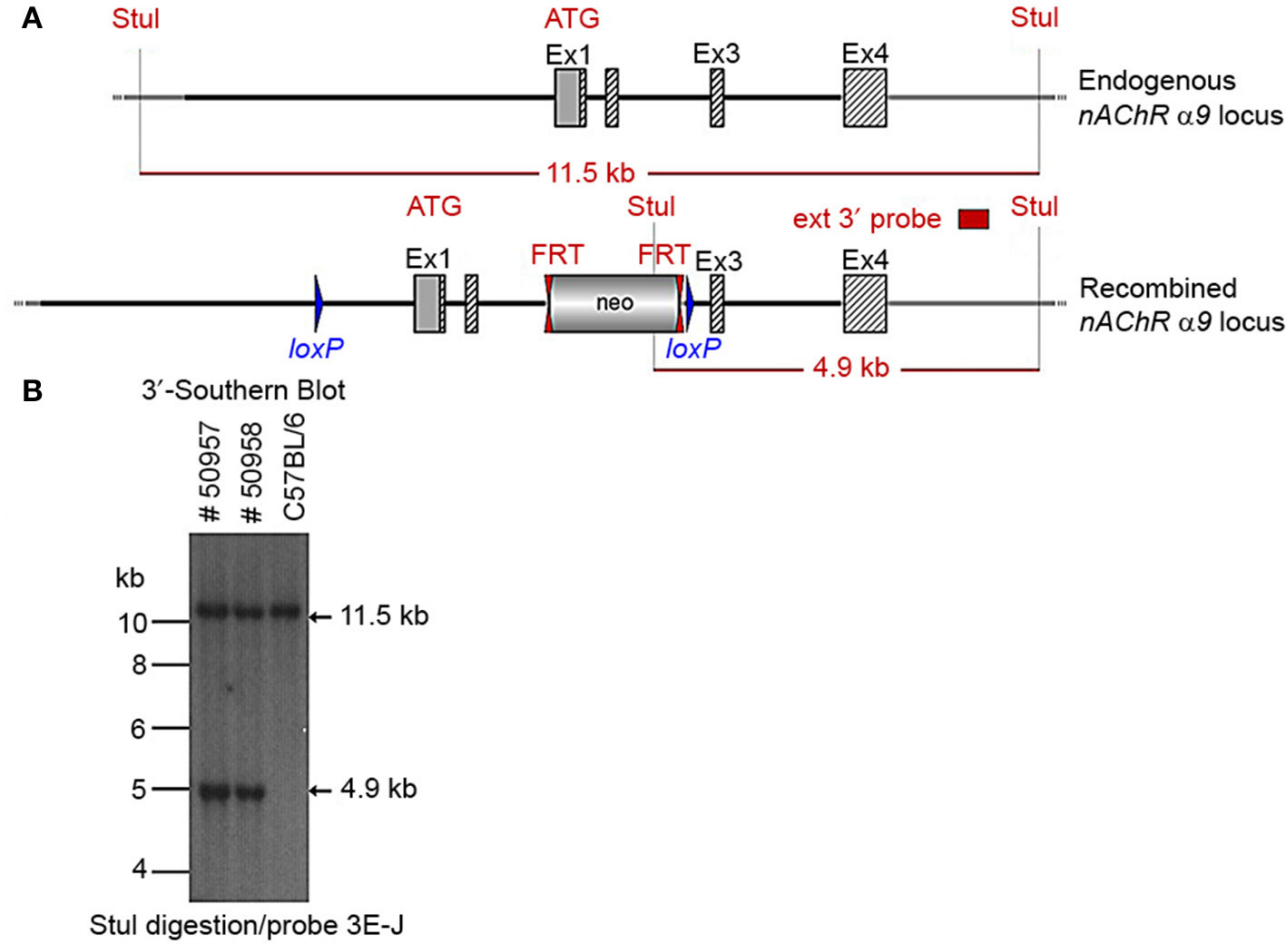
**(A)** The schemata for the endogenous and recombined a9 nAChR loci. **(B)** The Southern blot analysis of the α9 F1 generation. A 90% chimeric male mouse from clone HA11 was mated with C57BL/6J females. The genomic DNAs of two resulting agouti pups (#50957 and #50958) were compared to C57BL/6J wildtype DNA. StuI digested DNAs were blotted on nylon membrane and hybridized with the external 3′ probe (3E-J) to validate the heterozygosity of the nAChR α9 gene mutation.

Two clones produced surviving chimeras. Two male (agouti) chimeras were chosen for breeding to C57BL/6J animals to produce the F1 generation. Seven chimeras derived from one ES cell line transmitted the mutation successfully. One heterozygote male (50957) and one heterozygous female (50958) from the F1 generation were verified by Southern blot (Figure 3) and chosen for breeding.

**Figure 4:**
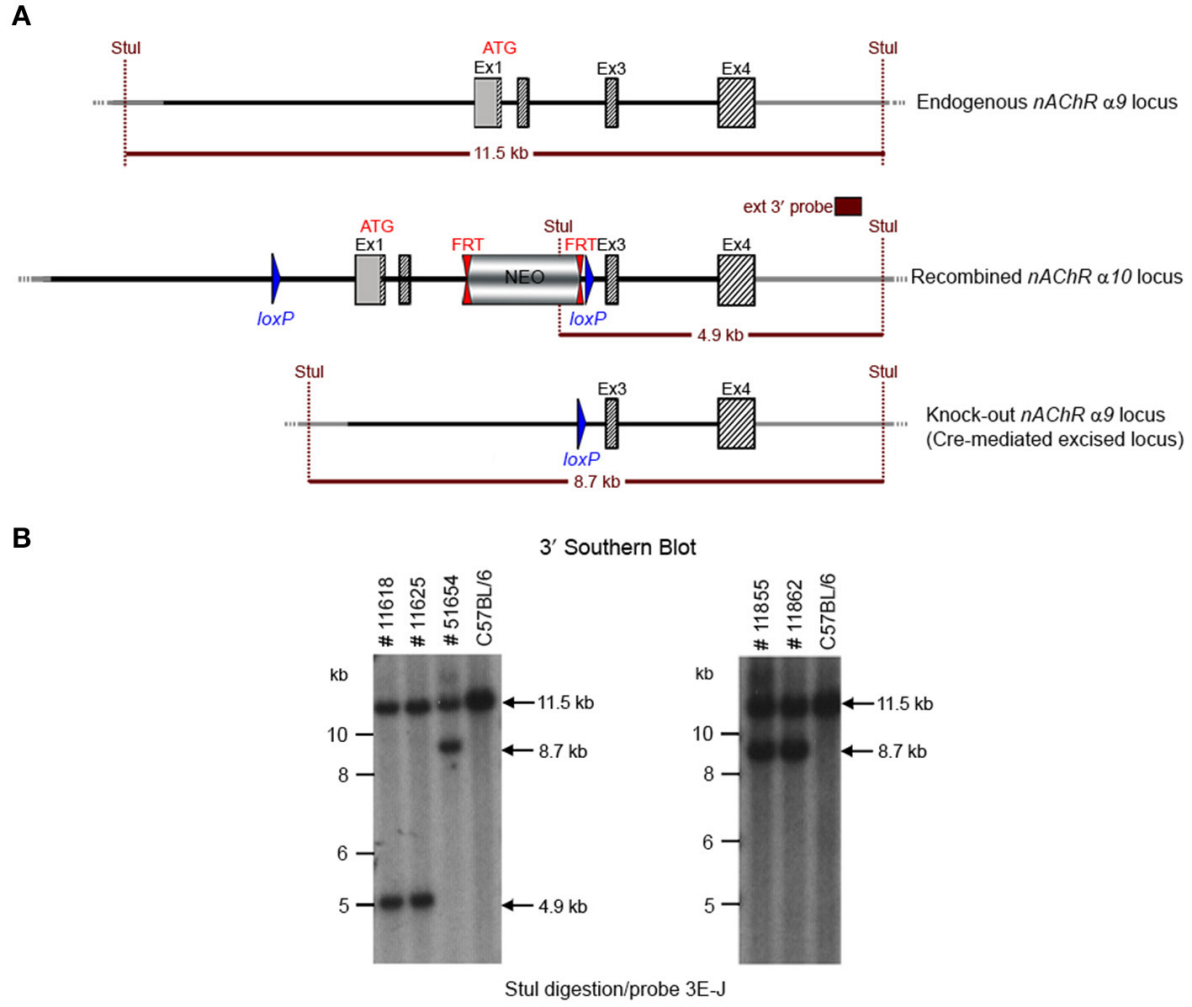
**(A)** The schemata for the endogenous and recombined a9 nAChR loci and the Cre-mediated excised locus. **(B)** The Southern blot analysis of the α9 N2 generation. Genotypes of pups derived from the N2 breeding with Cre delete mice was determined by PCR as described in the Methods section. To confirm the results of the PCR, the genomic DNA of five animals (#11625, #11618, #51654, #11855, and #11862) was tested by Southern blot. StuI digested DNAs were blotted on nylon membrane and hybridized with the external 3′ probe (3E-J). The constitutive knockout line was established from #11862. The conditional knockout line was established from #11625.

The floxed region (exons 1–2) was excised by mating the heterozygous F1 mice with C57BL/6J Cre deleter mice. The Cre-mediated excision event was confirmed by PCR using the primer sequences CCAGTCATAGCCGAATAGCCTCTCCAC/TGTCGAAGGGGAAATAGGTGACATCC to detect the recombination event. Three of the resulting pups from this N2 generation yielded an amplification product corresponding to the excised allele and no amplification product for a PCR-specific for the recombined non-excised allele, confirming the excision event, and indicating that they were heterozygous for the constitutive allele. Two heterozygous N2 mice and three N2 knockout animals were analyzed by Southern blot at the 3′ end (Figure 4) and their genotypes confirmed.

The potential founders were transferred to the Boys Town National Research Hospital (BTNRH). The constitutive knockout line was established from #11862 (see Figure 4). The conditional knockout line was established from #11625 (see Figure 4). The neomycin cassette was removed by breeding to C57BL/6J mice expressing FLP recombinase (Jackson Labs). Both the constitutive and conditional lines were backcrossed to >99% congenicity on the C57BL/6J background using MAXBAX (Charles River, Troy NY).

### Generation of the α10 conditional and constitutive mutants (knockouts)

The cloning and the initial development of the mice were conducted by Genoway, Inc. (Lyon, France). Twelve kb of mouse genomic DNA encompassing the α10 subunit gene region surrounding exons 1-3 on chromosome 7 were isolated from the 129Sv/Pas genetic background and fully sequenced.

**Figure 5:**
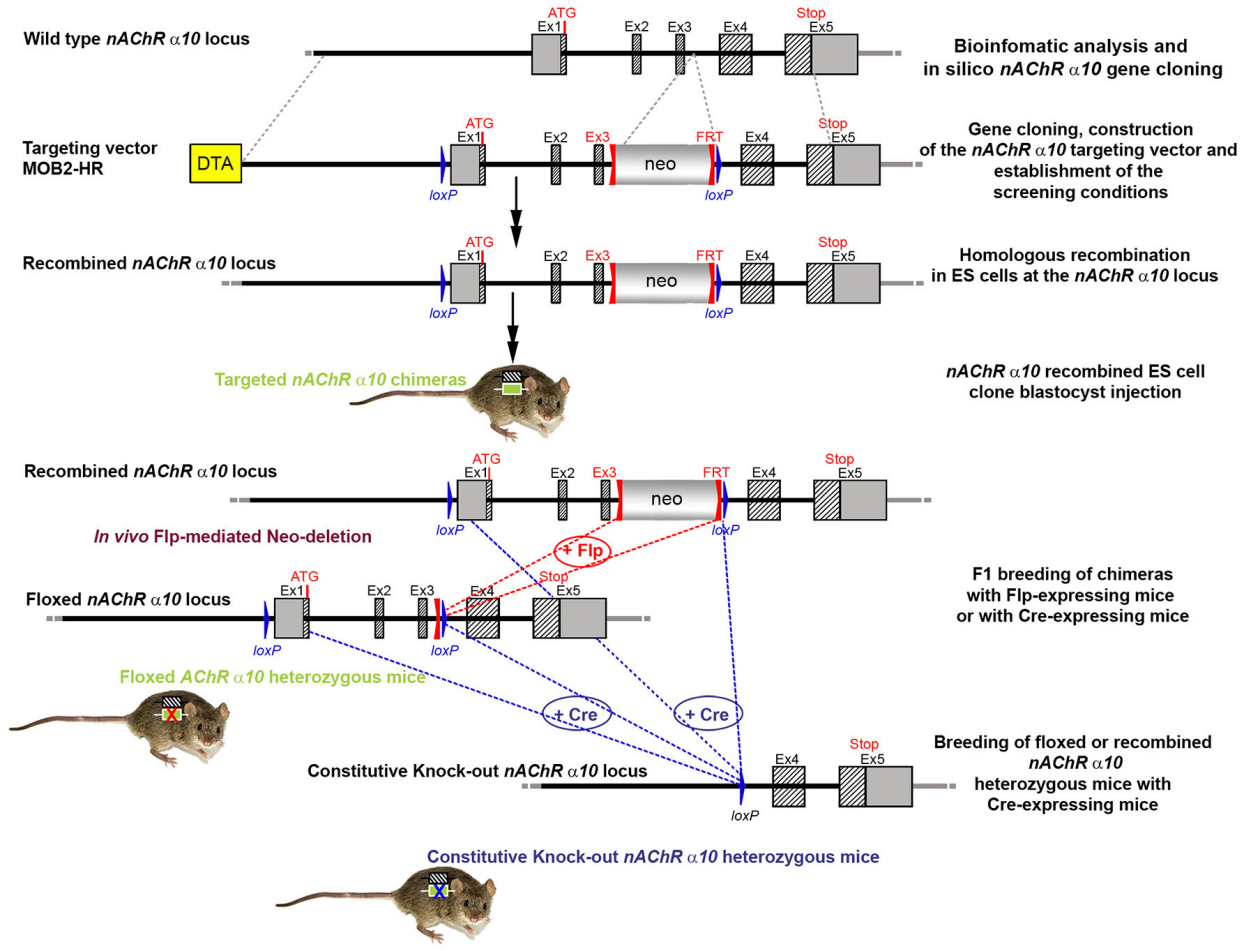
The targeting strategy for the generation of the *CHRNA10* knockout. Boxes represent exons. Solid line represents intronic sequences. LoxP and FRT elements are shown as blue and red triangles, respectively. Neo and DTA boxes represent the neomycin positive selection cassette and the Diphtheria Toxin A negative selection cassette. The constitutive KO was generated by breeding the floxed nAChR α9 heterozygous mice with Cre-expressing mice.

The targeting strategy for the generation of *CHRNA10* knockout is shown in Figure 5. Exons 1–3 of *CHRNA10* were deleted and their flanking intronic sequences were replaced by a validated FRT-neomycin-FRT-*loxP* cassette at the 3′ end and a single *loxP* site in the 5′ direction, which effectively eliminated the translation of any truncated form of the gene. The distal *loxP* site was positioned upstream of exon 1 within the promoter sequence.

A 7,866 bp positive control vector (MPB2-C+) and a 16,602 bp targeting vector (MOB2-HR) were constructed and validated. Neomycin (neo) was used as the positive selection marker. The targeting vector used the Diphtheria Toxin A (DTA) as a negative selection marker. Functionality of the *loxP* and FRT sites was confirmed by transformed *E. coli* with the targeting vector and PCR using the reverse primer TGACTCAGCTCACTCCCATGAAGACG and forward primer GCTGAGCGTCAAATGGGAAGGC, which were located downstream of the neomycin cassette and upstream of the *loxP* site, respectively.

**Figure 6:**
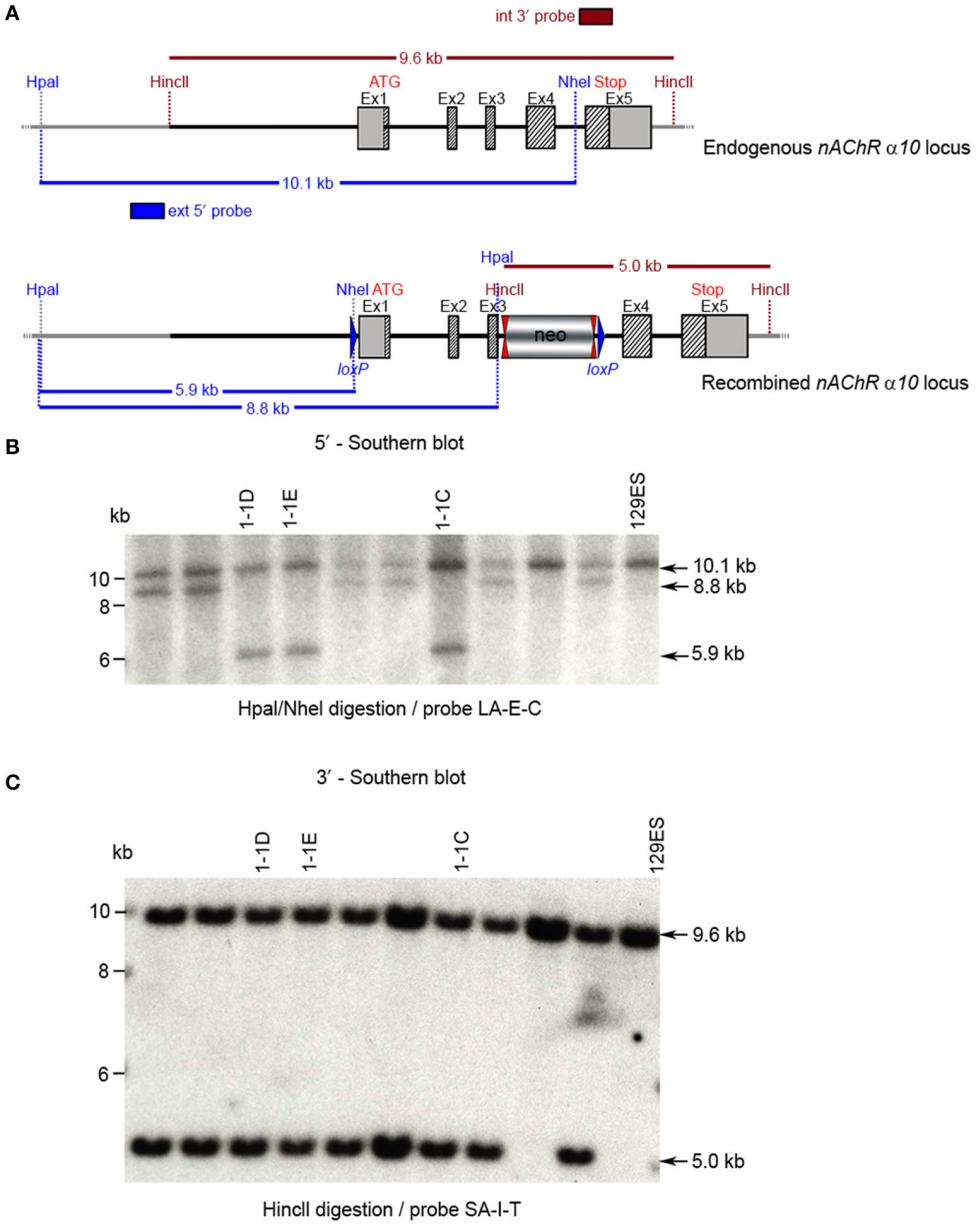
Southern blot analysis for 5′ and 3′ homologous recombination in ES cells (α10 nAChR). The genomic DNA of the 10 tested ES cell clones was compared with wild type DNA from 129ES. **(A)** Schematic representation of the WT and recombined α nAChR alleles with the relevant restriction sites for the Southern blot analysis. **(B)** DNA was digested with Hpal/Nhel and blotted on nylon membrane and hybridized the external 407 bp 5′ probe (LA-E-C) or **(C)** DNA was digested with HincII and hybridized to the internal 602 bp 3′probe (SA-I-T) to screen for the 5′ and 3′ homologous recombination. The external 407 bp 5′ probe was generated by PCR on genomic DNA using the primer pair CTCTGTTTTCCTGTAGGTGG/GAAGGTAGGACTGGGTAAGG. The 5′ probe (LA-E-C) hybridized downstream of the 5′ homology sequence of the targeting vector, which also allowed detection of the ES cell clones in which the targeting vector was integrated in a non-homologous manner. The 602 bp internal 3′ probe (SA-I-T) was generated by PCR on genomic DNA using the primer pair GCCCTTACTTTATCCTTCCCCACATCG/CAGCTCCCTGAAATCCAGTTCCTGG. The expected size of the WT allele was10132 bp and the recombined allele 5,922 bp, respectively, with the 5′ probe and 9,567 and 5,024 bp, respectively, with the 3′ probe. Cell clones 1-1D, 1-1E, and 1-3C all showed WT and recombined signals of similar intensity and were chosen for blastocyst injection.

**Figure 7:**
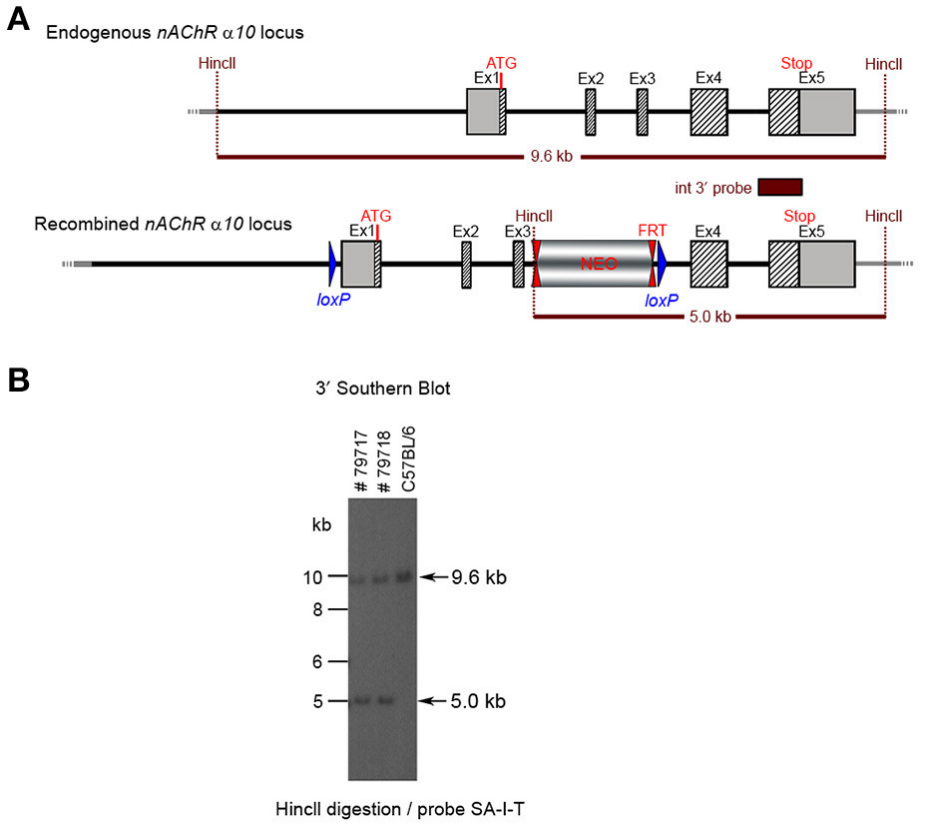
**(A)** The schemata for the endogenous and recombined a9 nAChR loci. **(B)** The Southern blot analysis of the α10 F1 generation. The genomic DNA of the two 80% chimeric F1 mice (#79717, #79718) from clone 1-1D was compared with C57BL/6J wildtype DNA. The HincII digested DNAs were blotted on nylon membrane and hybridized with the internal 3′ probe (SA-I-T) to validate the heterozygosity of the nAChR α9 gene mutation.

The targeting construct was linearized with Nrul and transfected into ES cells according to standard electroporation procedures. A total of 352 clones were isolated and 179 were screened with PCR at the 3′ end of the targeting vector using the primer sequences TGACTAGGGGAGGAGTAGAAGGTGGC/GGTTTGAGGTCAGAAGTGCTGGTCC. Ten clones were selected for analysis by Southern blot at both the 5′ and 3′ sides (Figure 6). Three clones were selected for blastocyst injection (see Figure 6). Three clones produced surviving six chimeric animals. Two male highly chimera mice were chosen for breeding to C57BL/6J females to produce the F1 generation. Three (agouti) females in the F1 generation carried the recombined allele. Recombination in two females (#79717 and #79718) was verified by Southern blot (Figure 7) and chosen for breeding.

**Figure 8:**
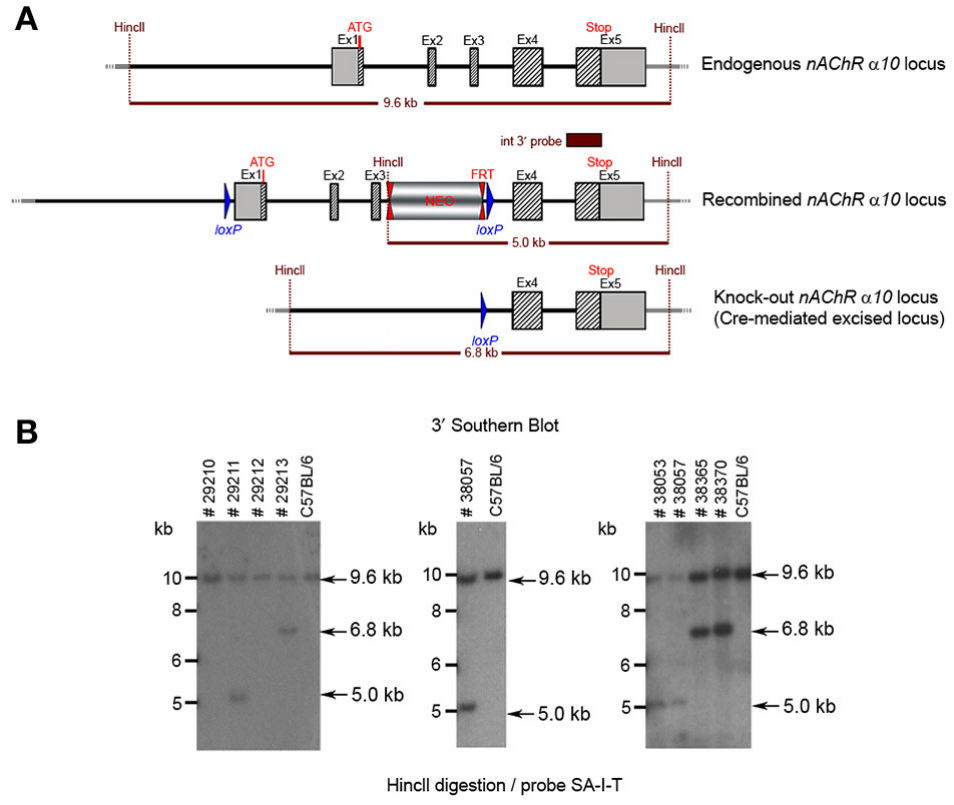
**(A)** The schemata for the endogenous and recombined a9 nAChR loci and the Cre-mediated excised locus. **(B)** The Southern blot validation of the α10 Heterozygous F1 or N2 generation. The genotype of the pups derived from the F1 or N2 breeding with Cre deleter mice was determined by PCR as described in the Methods section. To confirm the results of the PCR, the genomic DNA of eight animals: WT (#29210, #29212), recombined (#29211, #38053, #38057) and knockout (#29213, #38365, #38370). HincII digested DNAs were blotted on nylon membrane and hybridized with the internal 3′ probe (SA-I-T). The constitutive knockout line was established from #38370. The conditional knockout line was established from #38053.

The floxed region (exons 1–3) was excised by mating the heterozygous F1 mice with C57BL/6J Cre deleter mice. The Cre-mediated excision event was confirmed by PCR. Four mice from the F1 generation and 29 pups from the N2 generation were tested with PCR and yielded an amplification product corresponding to the excised allele, indicating that they were heterozygous for the constitutive allele. Southern blot analysis (Figure 8) confirmed the PCR results. Five male heterozygous recombined mice (#29211, #38057, #38059, #38366, #38049), four female heterozygous recombined mice (#38065, #38368, #38371, #38053), two male heterozygous knockouts (#38365, #38367) and two female heterozygous knockouts (#29213, #38370) and two incomplete excised heterozygotes were identified (#38051, #38056).

The potential founders were transferred to BTNRH. The constitutive knockout line was established from #38367 (see Figure 8). The conditional knockout line was established from #38368 (see Figure 8). The neomycin cassette was removed by breeding to C57BL/6J mice expressing FLP recombinase (Jackson Labs). Both the constitutive and conditional lines were backcrossed to >99% congenicity on the C57BL/6J background using MAXBAX (Charles River, Troy NY).

### Experimental animals

The physiological and morphological studies performed on wildtype (WT) and KO mice, which were all backcrossed to congenicity before use. Single knockouts were obtained from heterozygote females and either heterozygote or knockout males. Double knockouts were obtained from female mice heterozygote for α9 and α10 and male mice heterozygote for α9 and α10 or double α9α10 KO males. WT controls were from the same colony and obtained by mating heterozygote females with heterozygote or WT males or WT females with heterozygote males. The animals were bred and genotyped at BTNRH and shipped via air courier to the Kresge Institute or Washington University after weaning. Animals at BTNRH are free of all common pathogens.

All procedures for this study followed NIH guidelines and were approved by Institutional Animal Care and Use Committees (IACUC) of BTNRH, the Kresge Institute, and Washington University.

### PCR arrays

To confirm the absence of transcription in the knockout mice, a Qiagen (RRID:SCR_008539) custom RT^2^ Profiler™ PCR array (SA Biosciences, Valencia, CA) and the Applied Biosystems (Foster City, CA) 700 Sequence Detection System were used to quantify transcription of all nAChR subunits measured in the cochlear samples. Whole cochlear tissue from four WT, 4 α9 KOs and α10 KOs at age P10 were dissected in RNA *later*® (Life Technologies, Carlsbad, CA), and the tissue for each genotype was combined for processing. RNA was extracted and amplified using the recommended kits for the RT^2^ Profiler™ PCR arrays, including RNeasy, the RT^2^ First Strand kit, and RT^2^ SYBR green, all according to the manufacturer's instructions. We sequenced the resulting products identified in WT tissue to confirm that the primers specifically recognized α9 and α10. With this technique, it is not uncommon for primers to recognize irrelevant transcripts in the absence of the intended transcript, e.g., gene deletion. PCR products detected in the KO samples were also sequenced and found to be multiple irrelevant transcripts.

### Auditory brainstem responses

For auditory brainstem response (ABR) measurements, animals were anesthetized with a mixture of ketamine and xylazine (80/15 mg/kg, IP) and placed dorsally in a custom head-holder with an ES-1 freefield speaker (Tucker-Davis Technologies) seven cm directly lateral from the right ear. Subdermal platinum electrodes (Grass) were placed behind the right pinna (reference), on the vertex (active), and under the skin of the back (ground). A rectal probe was used to monitor temperature, which was maintained near 38°C using a DC current-based isothermal pad (FHC). Tonebursts 5 ms in duration (0.5 ms cos^2^ R/F) were presented 500–1,000 times at 20/s in descending intensity using a 5 dB minimum step size until wave I of the ABR could no longer be visually discerned. The stimulus level was then increased until the response re-appeared. Recording utilized Biosig32 and TDT hardware. ABR thresholds were obtained at 5, 10, 20, 28.3, 40, and 56.6 kHz, 1 day, and 2 weeks after post-exposure by an operator blinded to genotype.

### Distortion product otoacoustic emissions

For distortion product otoacoustic emissions recording, animals were anesthetized as for ABRs in a separate session. DPOAE recording was conducted using EMAV software (S. Neely, Z. Liu, BTNRH) in conjunction with TDT and custom hardware. F2 varied from 5 to 40 kHz; F1 frequencies were given by F2/1.2. L1 and L2 were held constant at 75 and 65 dB SPL, respectively, and calibrated in the ear canal using the calibration feature of EMAV. Stimuli were digitally synthesized at 200 kHz and output to the right ear using two TDT EC-1 speakers.

### Noise exposures

Broadband noise (4–45 kHz, 100 dB SPL) was produced and filtered with General Radio 1310 generators and KrohneHite 3550 filters, respectively. The spectral shape of the noise was as previously published (Ohlemiller et al., 1999). Two animals at a time were placed in a wired cage suspended between four speakers at 0, 90, 180, and 270 degrees azimuth in a single-walled sound-proof booth with foam treatment (Industrial Acoustics, Bronx, NY). The cage was rotated at 0.013 Hz during the exposure to achieve a homogeneous sound field. The overall noise level was measured offline at the center of the cage using a B&K 4135 ¼ inch microphone in a combination with a B&K 2231 sound level meter set to broadband (0.2–70 kHz).

### Efferent mediated adaptation

For efferent medicated adaptation of DPOAE experiments (Halsey et al., 2005) was performed on animals anesthetized with ketamine 65 mg/kg, xylazine 3.5 mg/kg, and acepromazine 2 mg/kg. Body temperature was maintained through the use of water circulating heating pads and heat lamps. Additional anesthetic (ketamine and xylazine) was administered if needed to maintain anesthesia depth sufficient to insure immobilization and relaxation.

The stimuli were generated and the response data collected using TDT System III hardware and a MATLAB™ script written in-house. Stimulus tones F1 (10,909 Hz) and F2 (13,083 Hz) were presented with a F2/F1 = 1.2 ratio, and the distortion product (2F1–F2) recorded at 8,736 Hz. Tones were presented via two EC1 drivers (TDT, aluminum-shielded enclosure made in house) connected through a microphone (Knowles Acoustics Itasca, IL electret condenser microphone, type FG-23329-P07, in custom fabricated coupler with custom fabricated amplifier). Responses for 12–14 F1 levels and 12 F2 levels for each F1 level (for a total of 144–168 L1/L2 combinations) were measured, starting with 1 dB intervals to localize the region of maximum adaptation, and 0.4 dB intervals in that region of maximum adaptation to fully map the region. For each level combination, primaries were presented in 1-s bursts, with 10 ms on and off ramps. A 2-s pause followed every primary tone presentation. Responses were collected with a sampling rate of 50 kHz. A fast Fourier transform (FFT) was performed on the response waveform with an analysis window of 25 ms, and the sound level of the distortion product was obtained for each window. Responses to four 1-s identical stimulus presentations were averaged.

### Immunocytochemistry

All animals were perfused transcardially with 4% paraformaldehyde, 0.1 M sodium phosphate, pH 7.4 for 5–10 min, and post-fixed 6–16 h at 4°C. Tissue samples were rinsed and stored in phosphate buffer until shipment to Baylor University for processing.

After dissection and isolation, each cochlea was decalcified, microdissected into half-turns and then incubated in 5% normal horse serum (NHS) with 0.3% Triton-X 100 in PBS for 1 h. This was followed by incubation overnight to 48 h in primary antisera. To label cholinergic efferent terminals, we used goat anticholine acetyltransferse (ChAT) at 1:200 (EMD Millipore, Chemicon, Tecumseh, CA; AB144P; RRID:AB_2079751). Hair cells were either labeled with rabbit anti-myosin VIIa (Proteus Biosciences: #25-6790; Ramona, CA) or stained with phalloidin (Alexa Fluor 488; ThermoFisher; #A12379; Grand Island, NY). The ChAT antibody was labeled with a 1 h incubation in a biotinylated secondary followed by 1 h in a streptavidin-conjugated Alexa Fluor (ThermoFisher, #P10994). The myosin VIIa antibody was labeled with a 1 h incubation in an Alexa Fluor secondary (ThermoFisher, #A11035). Cochlear lengths were obtained for each ear, and a cochlear frequency map computed to localize hair cells. Confocal z-stacks of the 8.0 and 32.0 kHz regions from each ear were obtained. Each stack spanned 80–100 μm of cochlear length, and two adjacent stacks were imaged at each locus. Images stacks were processed with Fiji Image Analysis software (Schindelin et al., 2012).

### Statistical analyses

The physiological data were analyzed with a one-way ANOVA and Dunnett's *post-hoc* individual comparisons between WT controls with each of the KO groups. To determine if there was an effect of age for the efferent adaptation studies, a linear regression analysis was conducted on each group and all animals combined. All statistical analyses were conducted using Prism 7 software (GraphPad, San Diego, CA).

## Results

### Generation of conditional and constitutive KO mice

The mouse *CHRNA9* gene is located on chromosome 5 and extends over 11-kb. It consists of 5 exons separated by 4 introns (GenBank XM_132045). The ATG translation initiation site and the Stop codon are located in exons 1 and 5, respectively. *CHRNA9* encodes for a 1,439 amino-acid open reading frame. There are no alternative splice variants or cryptic exons known for the mouse *CHRNA9* gene. The mouse *CHRNA10* gene (GenBank AK033068) is located on chromosome 7 and extends over 6-kb. It consists of 5 exons separated by 4 introns. The ATG translation initiation site and the Stop codon are located in exons 1 and 5, respectively. *CHRNA10* encodes for a 447 amino-acid open reading frame. There are no alternative splice variants or cryptic exons known for the mouse *CHRNA10* gene. *Nup98* is located 2.5 kb upstream of the α10 gene. *Art1* overlaps at the level of exons 4 and 5, but the targeting strategy was not expected to interfere with *Art1* gene regulation.

We generated conditional and constitutive KOs for the α9 and α10 subunits of the nAChR gene family on a congenic C57BL/6J background. Our targeting strategy removed exons 1 and 2 of the α9 gene and exons 1–3 in the α10 gene. Gene line transmission was confirmed by PCR and Southern blot analyses, as described in the methods section and in Figures 1–8.

**Figure 9:**
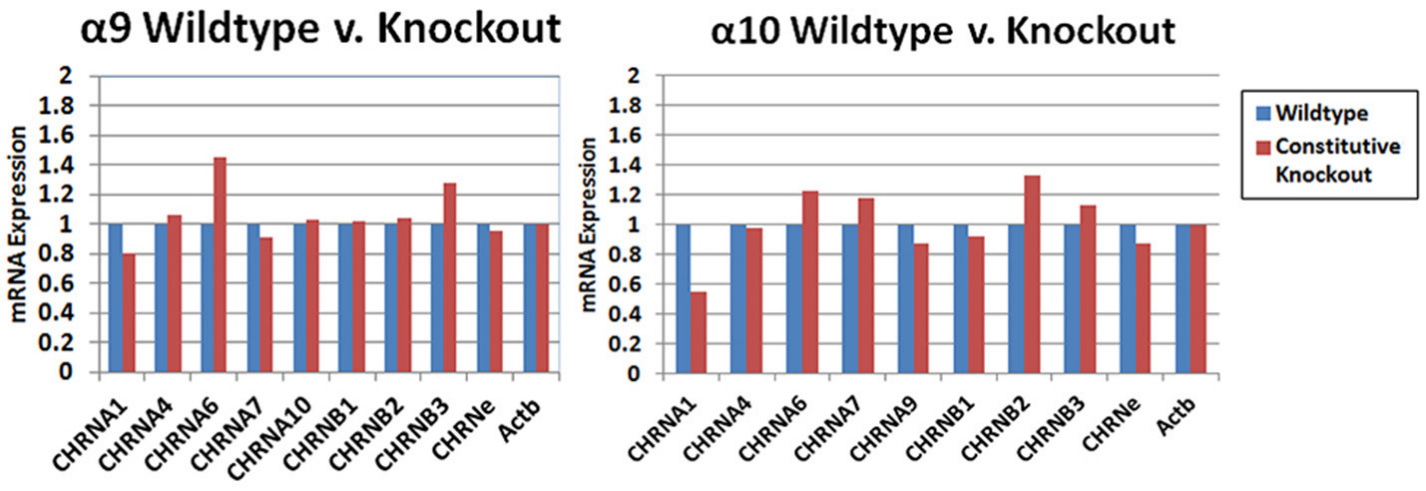
PCR arrays. Whole cochlear tissue was combined from four animals and the expression levels were determined with a custom Nicotinic Acetylcholine Receptor Qiagen RT^2^ Profiler™ PCR array. The transcripts for the α9 and α10 subunits in wild type mice were verified by sequencing. The small amount of product that was recognized by the primers in the KOs was also sequenced and were multiple irrelevant transcripts. There was no obvious change in transcription level of the other nAChRs that were detected in the cochlear tissues with this method.

The results of custom PCR arrays showed that the α9 and α10 transcription in cochlea of the constitutive α9 and α10 KOs, respectively, was eliminated and there was no apparent up-regulation by other nAChR subunits (Figure 9). In other publications, we reported that α9 transcript was absent in the retina of our α9 knockout (Smith et al., 2014), and that α9 and α10 transcription was absent in vestibular tissue of the respective constitutive KOs (Morley et al., 2017).

### ABR and DPOAE testing

**Figure 10:**
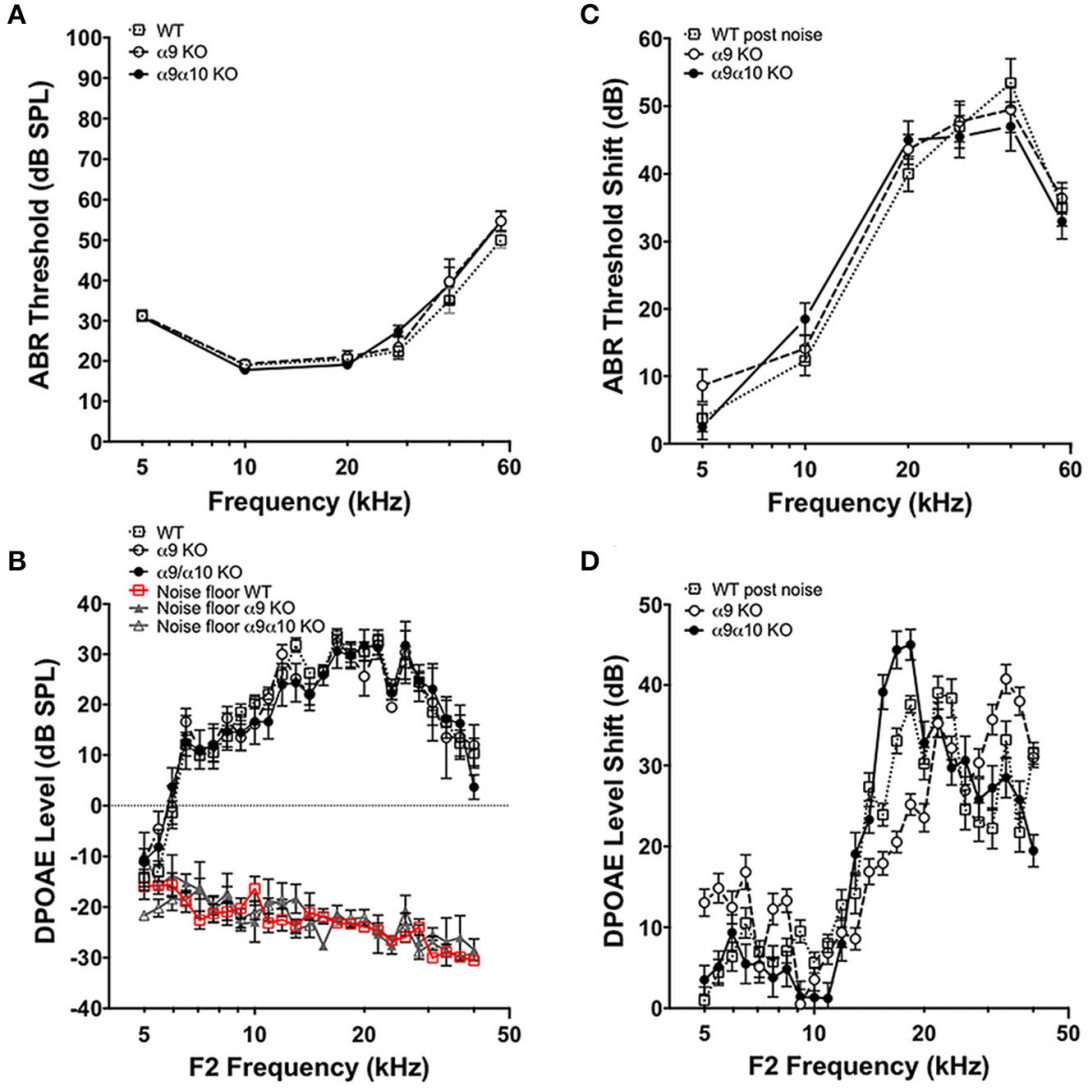
*In vivo* assays of hearing thresholds. **(A)** Plotted are the mean (±s.e.m.) ABR thresholds of pre-noise exposures for 2-month-old wild-type (WT, *n* = 23, gray open squares), α9 KO mutants (*n* = 11, white filled circles) and α9α10 KO mutants (*n* = 13, black filled circles). **(B)** ABR threshold shift (dB) at each frequency tested for wild-type (*n* = 18, gray open squares), α9 KO mutant (*n* = 11, white filled circles), and α9α10 KO mutant (*n* = 11, black filled circles) mice. **(C)** Mean (±s.e.m.) DPOAE levels as a function of f2 frequencies for 2-month-old wild-type (WT, *n* = 7, gray open squares), α9 KO mutants (*n* = 10, white filled circles), and α9α10 KO mutants (*n* = 8, black filled circles). The mean (±s.e.m.) noise floor for each genotype is also plotted for wild-type (WT, *n* = 7, red open squares), α9 KO mutants (n = 10, gray filled triangles), and α9α10 KO mutants (*n* = 8, gray open triangles). **(D)** Mean (±s.e.m.) DPOAE shift (dB) across all f2 frequencies for wild-type (gray open squares), α9 KO mutant (white filled circles), and α9α10 KO mutant (black filled circles) mice.

We performed tests of cochlear function on WT (*n* = 23), α9 KO (*n* = 11), and α9α10 double KO (*n* = 13) mice between 2–3 months old. ABR and DPOAE measurements in WT and KO mice were not statistically different for any of the frequencies measured (Figures 10A,C), consistent with previous studies suggesting that a dysfunction α9α10 nAChR does not alter cochlear sensitivities. There is evidence that the MOC efferent pathway provides some protection against noise-induced cochlear injury (Maison et al., 2013). The strength of MOC efferent protection and the physical number of the OHC nAChRs present in the inner ear both have been directly correlated with individual susceptibility to noise-induced hearing loss, whereas overexpression of the OHC efferent receptor has been shown to be protective against noise-induced hearing loss (Maison and Liberman, 2000; Luebke and Foster, 2002; Maison et al., 2002). We investigated whether our KO mice would show altered responses to noise. WT (*n* = 18), α9 KO (*n* = 11), and α9α10 double KO (*n* = 10) mice were exposed for 2 h to broadband noise (4–45 kHz) at 100 dB SPL. ABR and DPOAE measurements were repeated post noise exposure to assess protection from noise in the KO mice. After 2 weeks, ABR threshold shifts ranged from <10 dB at low frequencies and as high as 50 dB at higher frequencies. Similarly, DPOAE level shifts ranged from around 5–15 dB for low frequencies (5–10 kHz) and as high as 40–50 dB for higher frequencies. Overall, neither α9KO nor α9α10 KO mice showed any significant loss of protection from noise (see Figures 10B,D). Although ABR threshold shifts were similarly affected in all animals, DPOAE level shifts generated slightly varying patterns dependent on frequency. The greatest differences in DPOAE level shifts between genotypes were between 15 and 20 kHz. However, a one-way ANOVA for F2 frequencies between 15 and 20 kHz did not show any significant difference in DPOAE shift between WT, α9 KO, and α9α10 KO (*p* < 0.05).

### Efferent mediated adaptation

**Figure 11:**
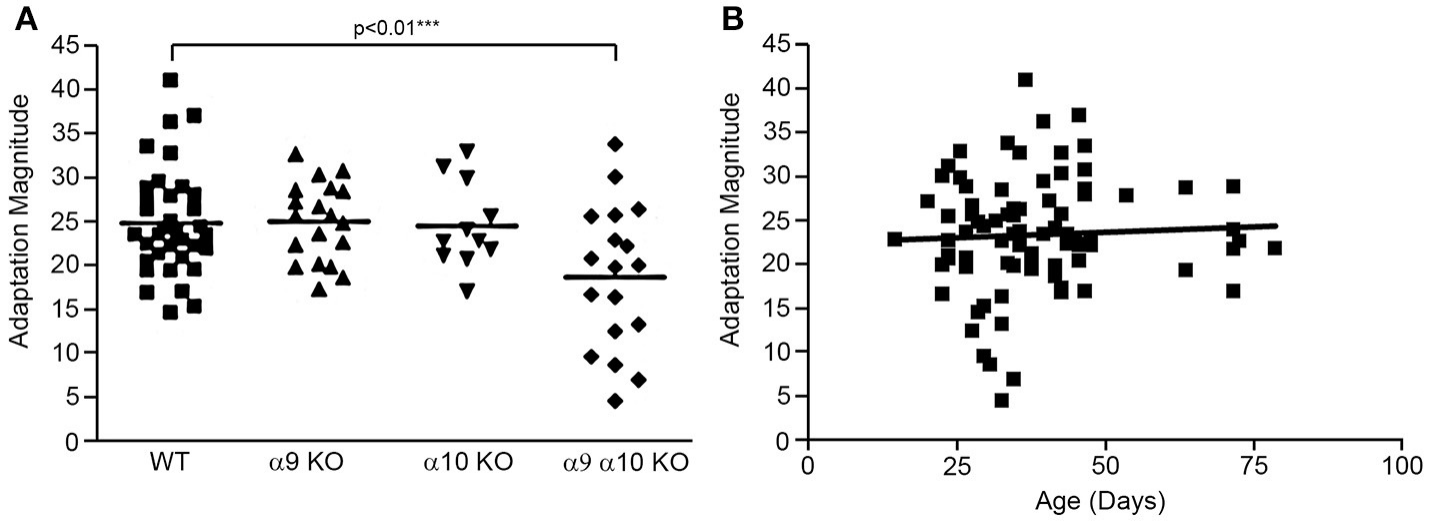
**(A)** Efferent mediated adaptation of DPOAE. Although there was a significant overall group effect [*F*_(3, 81)_ =4.33; *p* < 0.01], there was not a significant difference between wild type and either α9 or α10 knockouts. Only the double (α9α10) knockout group had significantly lower adaptation scores (*p* < 0.01). **(B)** The animals were tested as a young age (26–82 days) to avoid effects attributable to the *ahl* (age-related hearing loss) locus carried by C57BL/6J mice. There was not a correlation between adaptation scores and age in this study (*r* = 0.051).

Efferent-mediated adaptation of the DPOAE is a non-invasive measure of efferent function that has been shown to be a predictor of sensitivity to noise-induced trauma (Maison and Liberman, 2000; Halsey et al., 2005). Efferent mediated adaptation was determined in WT controls (*n* = 37), α9 KO (*n* = 19), α10 KO (*n* = 11), and α9α10 KO (*n* = 18) animals. Adaptation magnitude was similar for all groups, with the α9α10 KO mice having slightly higher values (Figure 11A). However, a one-way ANOVA showed that there was a significant difference between the WT controls and KOs [*F*_(3, 81)_ = 4.33, *p* < 0.01]. Using Dunnett's postdoc comparisons, only the α9α10 KO group was significantly different from controls (*p* < 0.01). Thus, the results of the efferent-mediated adaptation DPOAE experiments showed that neither the deletion of α9 or α10 subunits alone produced a significant decrement in efferent adaptation, but the deletion of both subunits did result in a significant decrement (Figure 11A). Anesthesia may attenuate the strength of the medical olivocochlear reflex (Chambers et al., 2012) and the differences between WT and KOs might be larger in anesthetized mice. The C57BL/6J strain carries an age-related hearing loss locus that affects hearing. Additionally, 1–3 month old mice show a developmental delay in suprathreshold sound-evoked activity of the auditory nerve (Dickerson et al., 2016). In order to show age was not related to the results obtained here, the animals were tested at a young age and efferent mediated adaptation scores were plotted as a function of age. A linear regression analysis was performed on each group and all of the animals combined. There was no significant relationship between age and adaptation magnitude for any group or the animals combined (*r* = 0.05). The combined regression plot is shown in Figure 11B.

### Efferent innervation patterns

**Figure 12:**
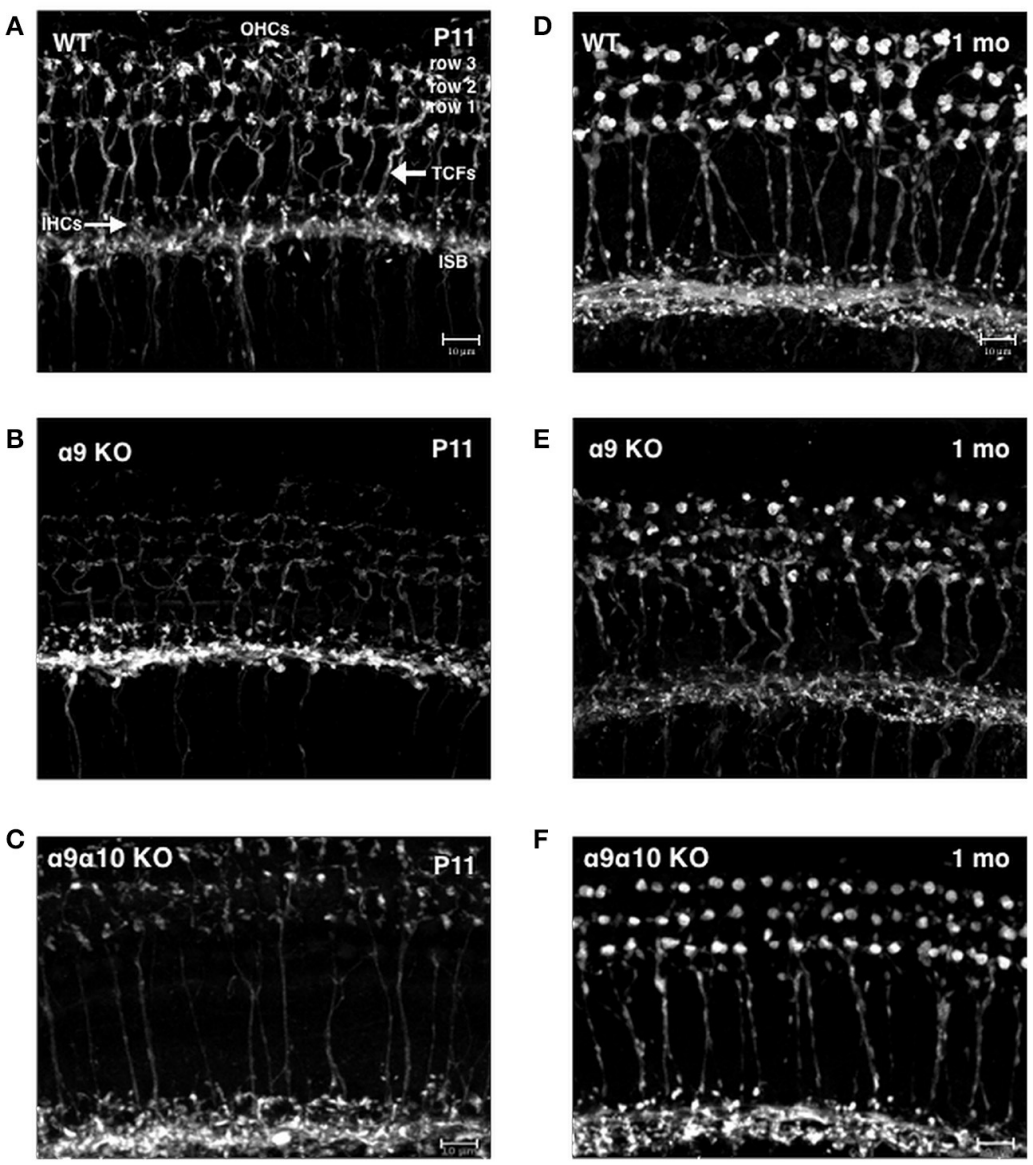
Confocal projections of cholinergic innervation patterns from middle regions (15–20 kHz) of the mouse cochlea. **(A)** In the wildtype (WT) animal at P11, ChAT-labeled efferent fibers and terminals are found below inner hair cells (IHCs) and in the inner spiral bundle (ISB). Thick ChAT-labeled efferent fibers cross the tunnel of Corti (TCFs) and terminals contact outer hair cells (OHCs) in rows 1, 2, and 3. **(B)** In the α9 KO mutant at P11, ChAT-labeling is more robust below IHCs but more sparse below OHCs. Very thin ChAT-labeled efferent fibers are seen crossing the tunnel of Corti. **(C)** In the α9α10 KO mutant at P11, ChAT-labeling is more robust below IHCs but more sparse below OHCs. Very thin ChAT-labeled efferent fibers are seen crossing the tunnel of Corti. **(D)** In the WT at 1 month, ChAT-labeling is found below IHCs in the ISB and large terminal clusters are seen on OHCs. ChAT-labeled efferent fibers cross the tunnel of Corti. **(E)** In the α9 KO mutant at 1 month, ChAT-labeling is found below IHCs. Thick ChAT-labeled efferent fibers cross the tunnel of Corti. Compared to WT, ChAT-labeled OHC terminal clusters are smaller with smaller individual terminals. **(F)** In the α9α10 KO mutant at 1 month, the ISB has ChAT-labeled fibers and terminals below IHCs OHC terminal clusters are smaller than WT but have larger individual terminals than in the α9 KO. ChAT-labeled efferent fibers cross the tunnel of Corti.

Previous studies of single α9 and α10 KOs show that the efferent innervation in the OHC region is abnormal (Vetter et al., 1999, 2007). We investigated whether the α9α10 KOs had abnormal innervation patterns that differed from the single subunit KO. We focused our investigation of the efferent innervation in α9 KO mice and α9α10 KO mice at two ages: postnatal days (P) 10–11 (just prior to the onset of hearing) and 1 month. In basal (high frequency) regions of the WT cochlea, efferent fibers and terminals travel in the inner spiral bundle (ISB) and give rise to tunnel crossing fibers (TCFs) that terminate as OHC efferent endings (Figures 12A,D). At P11, the ISB is a highly branched plexus with terminals surrounding the basal portions of inner hair cells (IHCs). The OHC efferent innervation in young postnatal animals consists of dense irregularly shaped and highly branched clusters of cholinergic terminals (Figure 12A). In the 1-month old WT, the ISB is a densely labeled bundle with fewer terminals surrounding IHCs. The cholinergic terminals below OHCs are regularly arrayed by OHC row, larger terminal cluster sizes (Figure 12D). The cholinergic innervation differed substantially in the single and double KO at both ages (Figures 12B,C,E,F). In the α9 KO at P11, cholinergic terminals and fibers are intensely labeled in the ISB, however, TCFs are thin and OHC efferent terminals are weakly labeled with small terminals compared to WT. In the α9α10 KO at P11, the ISB is also intensely labeled and TCFs were thinner and fewer in number than in WT. There were also fewer cholinergic terminals and the size of the terminal clusters was much smaller than in WT mice. At 1 month, the ISB in α9α10 was similar to WT as well as the OHC terminals were highly arrayed. Unlike WT, the terminal cluster sizes were smaller and more similar to the α9 KO.

## Discussion

The knockout models for the α9 and α10 nAChR subunits on a C57BL/6J background will have wide application for the study of their function in diverse tissues. The mouse models were generated by deletion of exons 1–2 in the α9 and 1–3 in α10, and their associated introns, eliminating activity and proper folding in both genes. The strains were backcrossed onto a C57BL/6J background because it is the most common genetic background used for mouse models, and would therefore allow the easy development of double or triple KOs. The constitutive KOs generated by crossing α7, α9, and α10 are viable and fertile.

In this manuscript we reported the auditory characterization of the α9, α10, and α9α10 double knockouts. The α9α10 nAChR mediates the neurotransmission of the MOC efferent system. The MOC system innervating the OHCs constitutes a sound-evoked reflex pathway that is excited by sound in either ear (Folsom and Owsley, 1987; Liberman, 1989). In all species investigated, activation of the MOC system decreases cochlear sensitivity. In the present study, the most notable finding is that the deletion of both α9 and α10 subunits was required before a significant decrement was observed in efferent strength, i.e., the magnitude of the loss of cochlear sensitivity. The results suggest that neither subunit alone is capable of maintaining efferent activity in this paradigm.

The results are somewhat at odds with previous data obtained using a α9 KO generated by the deletion of exon 4 (and its flanking intronic sequences) and backcrossed onto a mixed CBA/CaJ X 129Sv background. In initial studies, it was concluded that the deletion of α9 was sufficient to render the α9α10 nAChR incapable of carrying current (Vetter et al., 2007; Taranda et al., 2009). Subsequent studies comparing α9 KOs with WTs (e.g., Mohammadi and Christie, 2014; Terreros et al., 2016; Mohammadi et al., 2017; Tu et al., 2017), have typically not used α10 KO mice, but presumed that deletion of α9 is equivalent to a deletion of the α9α10 receptor. Thus, any contribution of α10 would not have been detected.

The results reported here, however, are consistent with our studies of the vestibular system in our mouse models. We recently reported the analysis of vestibular sensory evoked potentials (VsEPs) in α9, α10, α9α10, α7, and α7α9 knockouts (Morley et al., 2017). The results were complex. As in the experiment reported here, however, the most affected phenotype was the α9α10 double KO; the α9 single KO did not recapitulate the α9α10 KO. Unexpectedly, the α10 KO had a phenotype different from the WT, α9, or the α9α10; moreover, the α10 KO phenotype was similar to the α7 KO, indicating that perhaps α7 and α10 could be assembled in cells relevant to the vestibular phenotype. Co-expression and spatial localization of α7 and α10 was previously reported in the sympathetic neurons (Lips et al., 2006).

In other reports, we reported the phenotypes of the α9, α10, α9α10, and α7α9 knockouts in an Experimental Autoimmune Encephalitis (EAE) model of Multiple Sclerosis using our KO models (Simard et al., 2013; Liu et al., 2017). There was no difference between α9 and α9α10 KOs in the EAE model, but the α7α9 differed from the α7 or α9 KO.

The disorganization of the cholinergic MOC innervation in the cochlea observed in our mouse models is similar to that reported by others in α9 and α10 KOs (Vetter et al., 1999, 2007). We did not quantify the results and therefore any subtle differences would not have been detected. At least at the gross level, however, there were some minor morphological differences in the efferent innervation between the α9, α10, and α9α10 KO and all differed from WT suggesting that different combinations of subunit loss may be capable of slight alterations in the development of efferent innervation patterns.

Differences in the physiology (this report; Morley et al., 2017) between our α9 and α10 constitutive KO mice and the models first described by Elgoyhen et al. (1994, 2001) may be attributable to genes in the background strains that could modify the phenotypes. Unlike earlier lesioning experiments, behavioral thresholds of the previous α9 KO model did not show any performance deficits in the presence of background noise (May et al., 2002). It is also now known that α9 and α10 are among the nAChRs that mediate metabolic signaling (Richter et al., 2016; Backhaus et al., 2017; Zakrzewicz et al., 2017). Epigenetic differences may also be a factor; the α10 gene has CpG islands within the body of the gene (although α9 does not). Few laboratories control genetic drift, many do not maintain pathogen-free laboratories, and the environmental noise is not always monitored. These are factors, usually unknown and/or unreported, that can affect phenotypes. Finally, we expect that double and multiple KOs and conditional KOs will shed light on the complex relationship among nAChRs in many systems.

## Author contributions

BM participated in the generation and development of the animals models, research design, performance of experiments, interpretation of the data, and writing of the manuscript; DD and KO conducted the electrophysiology experiments and participated in research design, performance of experiments, interpretation of the data, and writing of the manuscript; DS conducted the confocal experiments, participated in research design, performance of experiments, interpretation of the data, and writing of the manuscript.

### Conflict of interest statement

The authors declare that the research was conducted in the absence of any commercial or financial relationships that could be construed as a potential conflict of interest.
